# A Case-Based Report on Prosthetic Management of Anterior Tooth Loss in Children: Developmental vs. Acquired Causes

**DOI:** 10.7759/cureus.91899

**Published:** 2025-09-09

**Authors:** Hulya Cerci Akcay, Cagan Tas, Eda Sir

**Affiliations:** 1 Pediatric Dentistry, Kocaeli Health and Technology University, Kocaeli, TUR; 2 Dentistry, Kocaeli Health and Technology University, Kocaeli, TUR

**Keywords:** anterior tooth loss, early childhood caries, pediatric dentistry, prosthetic rehabilitation, tooth agenesis

## Abstract

Anterior tooth loss in children, whether arising from developmental anomalies such as congenital agenesis or acquired conditions like early childhood caries, can compromise oral function, facial esthetics, and psychosocial well-being. This case report presents the prosthetic rehabilitation of two pediatric patients with distinct etiologies of anterior tooth loss. In one instance, a removable partial denture was fabricated to restore function and appearance following the caries-related loss of maxillary incisors, while in the other, a lingual arch incorporating a single-tooth pontic was employed to preserve space and esthetics in a child with bilateral agenesis of mandibular central incisors. Both treatment approaches were individualized according to the patient’s growth stage and clinical requirements, resulting in favorable outcomes in terms of oral function, esthetic improvement, and psychosocial adaptation.

## Introduction

Anterior tooth loss in children is a prevalent clinical condition with multifactorial origins, typically categorized as either developmental or acquired in nature [1]. Developmental causes often include congenital anomalies such as tooth agenesis, which is characterized by the absence of one or more teeth due to disturbances in the early stages of odontogenesis. Tooth agenesis can be classified according to severity: anodontia, defined as the complete absence of all teeth, and oligodontia, the absence of six or more teeth, excluding third molars [2]. This condition can occur in isolation or as part of complex syndromes and has been reported to affect both the primary and permanent dentitions, with prevalence rates ranging from 0.1% to 2.4% in the primary dentition and up to 7% in the permanent dentition [3]. Acquired tooth loss, on the other hand, is frequently attributed to early childhood caries (ECC), a highly aggressive and multifactorial disease that remains one of the most common chronic conditions in pediatric populations worldwide [4]. ECC can lead to premature loss of maxillary anterior teeth, often before the eruption of successors, and poses significant challenges in functional and psychosocial development [5].

Anterior teeth play a pivotal role not only in mastication and phonetics but also in guiding the proper eruption of permanent successors, supporting the perioral musculature, and maintaining the child’s facial esthetics and self-esteem [6]. Their premature loss-whether congenital or acquired-may result in space loss, drifting of adjacent teeth, disruption of occlusal harmony, and altered facial growth patterns [7]. Consequently, the absence of anterior teeth during critical phases of craniofacial development necessitates prompt intervention to prevent long-term functional and esthetic complications [8].

Prosthetic rehabilitation in children must be tailored to the etiology of tooth loss, the stage of dental development, and the child’s overall cooperation and psychosocial readiness [9]. Treatment options range from removable partial dentures to fixed space maintainers and, in selected cases, implant-supported restorations after growth completion [10]. Early prosthetic intervention aims to restore oral function, maintain space for permanent successors, improve esthetics, and enhance the child’s quality of life [11].

This case report presents two children with anterior tooth loss due to distinct etiologies: one involving bilateral congenital agenesis of the mandibular central incisors, and the other resulting from ECC-related maxillary incisor loss. The report aims to highlight the critical role of etiology-specific prosthetic management, the clinical decision-making process, and the functional and psychological outcomes of individualized treatment strategies during the mixed dentition period.

## Case presentation

Case descriptions with methodology

Case 1: ECC-Related Anterior Tooth Loss in a 5-Year-Old Male Patient

A five-year-old male patient was referred to the pediatric dentistry clinic with the chief complaint of compromised esthetics and impaired oral function due to extensive decay in the maxillary anterior region. Clinical examination revealed severe destruction of the primary maxillary central and lateral incisors (teeth #52, 51, 61, and 62), with pulpal involvement and dark brown discoloration. Additionally, the maxillary right and left first primary molars (teeth #54 and 64) had been previously extracted due to caries-related complications. No relevant medical or family history was reported.

Based on the clinical findings and parental expectations, a treatment plan was formulated to fabricate a removable functional space maintainer with esthetic anterior elements. Under local anesthesia, the remaining maxillary anterior teeth were extracted, and soft tissue healing was monitored over two weeks. A removable prosthesis with an acrylic base and prefabricated denture teeth was designed to restore esthetics and maintain arch length. Circumferential clasps were applied on the primary canines (#53 and #63), and Adams clasps on the second primary molars (#55 and #65) to ensure adequate retention and stability.

**Figure 1:**
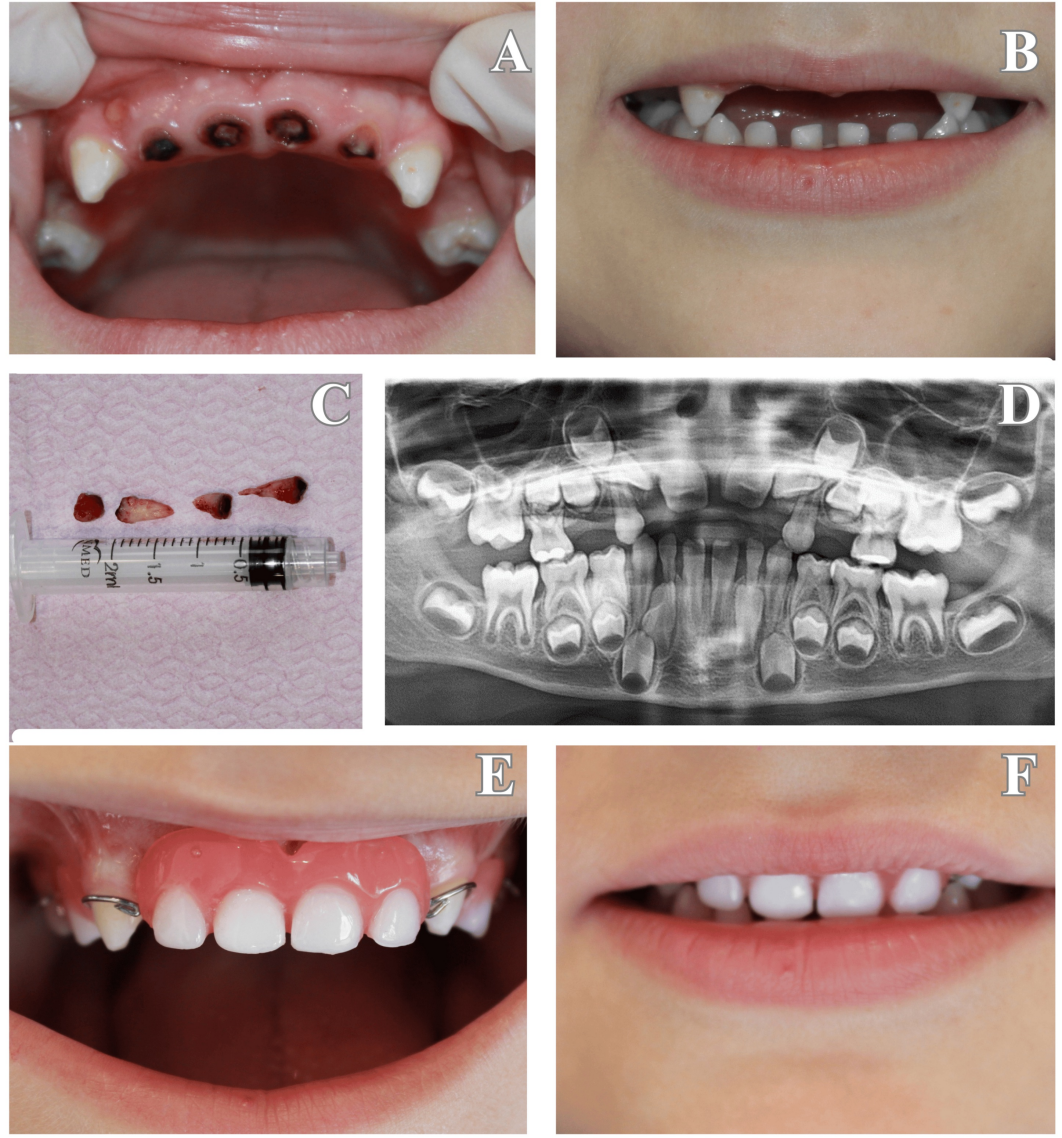
(A) Intraoral view showing extensive carious destruction and pulpal involvement of the maxillary primary incisors. (B) Preoperative frontal view of the patient demonstrating esthetic and functional compromise. (C) Extracted teeth (#52, 51, 61, 62, 54, and 64) due to severe caries-related complications. (D) Panoramic radiograph revealing multiple missing primary teeth and the stage of development of the permanent successors. (E) Intraoral view after the delivery of a removable functional space maintainer with esthetic anterior elements. (F) Extraoral view showing improved esthetics and smile harmony after appliance placement.

Following appliance insertion, detailed instructions on oral hygiene, appliance care, and wear duration were provided to both the child and caregivers. At the one-month follow-up visit, the patient exhibited satisfactory adaptation to the prosthesis, with improved facial esthetics, no mucosal irritation, and normalized speech and mastication. The overall treatment process, including preoperative findings, extractions, prosthetic appliance design, and follow-up outcomes, is summarized in Figures 1A-1F.

Case 2: Bilateral Agenesis of Mandibular Central Incisors in a 9-Year-Old Male Patient

A 9-year-old male patient presented to the pediatric dentistry department with concerns regarding anterior spacing and esthetics in the mandibular region. Intraoral and panoramic radiographic evaluation confirmed bilateral agenesis of the permanent mandibular central incisors (teeth #31 and #41), with no associated trauma, systemic illness, or family history of hypodontia. In the differential diagnosis, delayed eruption was excluded based on the absence of radiographic evidence of tooth germs, which confirmed the agenesis diagnosis.

**Figure 2:**
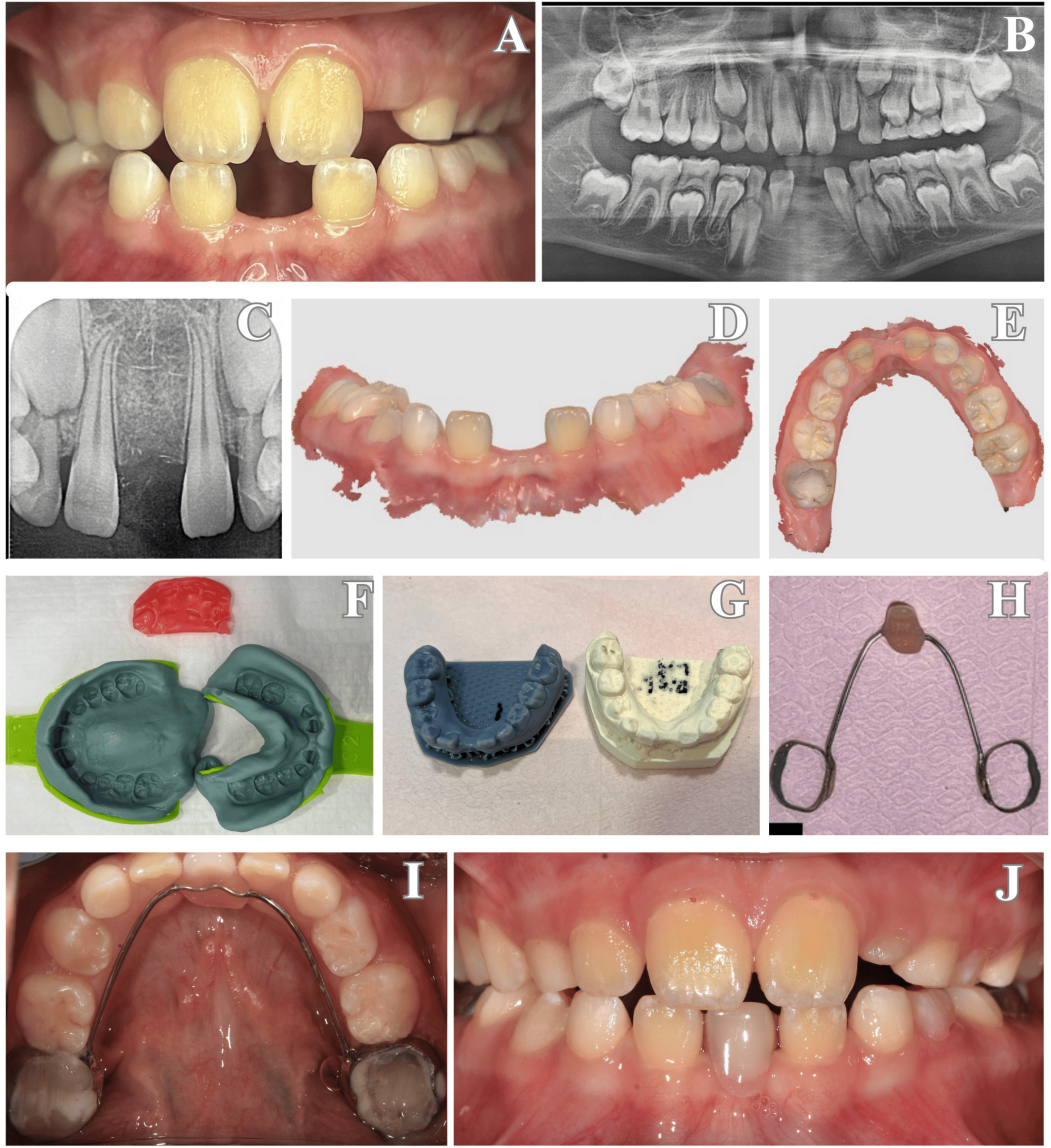
(A) Intraoral view showing the congenital absence of the mandibular permanent central incisors. (B) Panoramic radiograph confirming bilateral agenesis of teeth #31 and #41. (C) Periapical radiograph for further evaluation of the anterior mandibular region. (D, E) Digital impressions and 3D scan images for appliance planning. (F) Conventional impression using type A silicone and wax bite registration. (G) Working model obtained from the impressions. (H) Fabricated single-tooth pontic integrated into a mandibular lingual arch appliance. (I, J) Post-insertion intraoral views of the final appliance in occlusion and from the occlusal perspective.

To maintain arch integrity and prevent mesial drift of adjacent teeth, a customized lingual arch appliance was planned (Figures 2A-2J). A single-tooth pontic was incorporated to address the esthetic deficiency. The appliance was fabricated and cemented onto the permanent mandibular first molars (#36 and #46) using glass ionomer cement. The adaptation period was approximately 2 weeks, with no reported soft tissue irritation. The patient was followed periodically to evaluate growth, space maintenance, and the eruption status of adjacent teeth.

Preventive dental care, including fluoride applications and dietary counseling, was provided. Future orthodontic and prosthodontic treatment alternatives were discussed with the family, taking into consideration the child’s age, skeletal development, and psychosocial needs.

## Discussion

Anterior tooth loss in pediatric patients poses a significant clinical challenge due to its adverse impact on oral function, esthetics, phonetics, and psychosocial development [10]. The etiology of tooth loss plays a crucial role in determining the appropriate treatment modality, and early diagnosis is essential for guiding timely intervention [2]. In the present report, two pediatric cases with distinct etiologies - ECC and congenital agenesis of mandibular central incisors - were managed with tailored prosthetic approaches to meet the functional and esthetic needs of the developing child.

In ECC-associated tooth loss, rapid destruction of primary anterior teeth frequently leads to premature extractions, resulting in compromised mastication, speech development, and appearance [12-14]. In the case of the five-year-old male patient presented here, a removable partial denture was selected as a transitional solution. This modality was chosen to maintain arch integrity, restore function and esthetics, and support psychosocial adaptation during the mixed dentition period [15]. As supported by literature, removable appliances offer flexibility, ease of adjustment, and are well tolerated by pediatric patients when regular follow-up and caregiver compliance are ensured [10,14]. Moreover, early prosthetic rehabilitation following ECC has been shown to enhance oral health-related quality of life and prevent adverse behavioral outcomes [16].

Conversely, the second case addressed a non-syndromic 9-year-old patient with bilateral agenesis of the permanent mandibular central incisors - a less common but clinically significant developmental anomaly. Anterior hypodontia can compromise occlusal stability, phonetic clarity, and midline symmetry. In accordance with contemporary guidelines, a passive lingual arch appliance incorporating a single-tooth pontic was employed to preserve space, support esthetics, and allow for future definitive prosthetic or orthodontic planning [17,18]. This approach is especially advantageous in growing patients, as it maintains dental arch integrity without impeding skeletal development and allows modifications in harmony with the child’s maturation. A summary of developmental versus acquired anterior tooth loss and their respective management strategies during the mixed dentition period is provided in Table 1, highlighting the clinical, diagnostic, and psychosocial considerations that guide individualized treatment planning.

**Table 1:** Comparison of developmental versus acquired anterior tooth loss and their management during the mixed dentition period.

Etiology	Clinical Features	Diagnostic Considerations	Management Strategy	Functional & Psychological Outcomes
Developmental (Congenital Agenesis of Mandibular Central Incisors)	Absence of the tooth germ; no eruption in the expected period	Radiographic absence of the tooth bud confirms agenesis, excluding delayed eruption	Removable partial prosthesis or space maintainer to preserve esthetics and function	Restores oral function and appearance; improves self-confidence and psychosocial adaptation
Acquired (ECC-related Maxillary Incisor Loss)	Loss of erupted teeth due to extraction following caries or infection	Radiographic confirmation of extraction socket; assessment of arch space	Functional space maintainer or prosthetic replacement, depending on the extent of loss	Prevents space loss and malocclusion; supports esthetics, speech, and psychosocial well-being

The treatment plans in both cases reflect a multidisciplinary, evidence-based approach focused on growth monitoring, functional rehabilitation, and psychosocial well-being [19]. As emphasized by Goswami and Chauhan and Kaikure et al., prosthetic intervention during early childhood plays a critical role in enhancing self-esteem and facilitating social integration, especially in cases with visible anterior tooth loss [10,12]. Regardless of appliance type-removable or fixed-regular reevaluation is essential to accommodate craniofacial changes and anticipate long-term needs, including orthodontic alignment or implant-based rehabilitation in adolescence [20,21]. Alternative options, such as fixed partial dentures or implant-supported restorations, were not suitable at this age due to ongoing craniofacial growth. Transition to permanent prosthetic rehabilitation will be planned once skeletal growth stabilizes.

Collectively, these cases underscore the importance of individualized prosthetic strategies in pediatric dentistry. Successful outcomes depend on timely diagnosis, careful appliance selection, interdisciplinary coordination, and continuous follow-up throughout the child’s developmental trajectory.

Patient perspective

From the parents’ perspective, the treatment approach was considered satisfactory in terms of restoring esthetics and function. Moreover, they emphasized that individualized prosthetic management during the mixed dentition period contributed positively to their children’s psychological adaptation and social interactions.

## Conclusions

Early and etiology-specific prosthetic rehabilitation is crucial for preserving oral function, maintaining arch integrity, and supporting psychosocial well-being in pediatric patients with anterior tooth loss. The present cases demonstrate that individualized management strategies can provide favorable short-term outcomes; however, these results should be interpreted cautiously, as long-term adaptation may vary depending on growth, patient cooperation, and family support. Continuous follow-up and timely transition to definitive prosthetic or orthodontic solutions remain essential for ensuring stable long-term oral health and functional outcomes.
